# Combination of chest CT and clinical features for diagnosis of 2019 novel coronavirus pneumonia

**DOI:** 10.1515/med-2020-0107

**Published:** 2020-07-28

**Authors:** Xin Wang, Peng Wen, Zhi-Gang Sun, Chun-Yan Xing, Yun Li

**Affiliations:** Department of Respiration, Jinan Central Hospital, Cheeloo College of Medicine, Shandong University, Jinan 250013, People's Republic of China; Department of Respiratory Medicine, Shandong Provincial Chest Hospital, Cheeloo College of Medicine, Shandong University, Jinan 250013, People's Republic of China; Department of Thoracic Surgery, Jinan Central Hospital, Cheeloo College of Medicine, Shandong University, Jinan 250013, People's Republic of China; Department of Intensive Care Unit, Jinan Central Hospital, Cheeloo College of Medicine, Shandong University, Jinan 250013, People's Republic of China

**Keywords:** coronavirus pneumonia, COVID-19, CT, nucleic acid test

## Abstract

In December 2019, novel coronavirus pneumonia-19 (COVID-19) was discovered in the viral pneumonia cases that occurred in Wuhan, China, and then quickly spread around the world. This report described the clinical course of two COVID-19 patients and the purpose of the study was to discuss the combination of chest CT and clinical features for diagnosis of COVID-19. The first case was a typical COVID-19 case. A 66-year-old female presented to our hospital with a 3-day history of fever. She had contact with a COVID-19 patient. Chest CT showed a typical COVID-19 appearance. She was diagnosed with COVID-19 by a positive nucleic acid test. The second case was a 50-year-old male with a 2-day history of fever. He denied having been to Wuhan. Chest CT also showed typical features of COVID-19 pneumonia. COVID-19 nucleic acid tests were repeated up to seven times and the results remained controversial. Eventually, he was diagnosed with COVID-19. Our study shows that chest CT has high sensitivity for diagnosis of COVID-19 in clinical practice, particularly when the nucleic acid test is negative. The chest CT should be considered as a diagnostic tool for the COVID-19 screening, comprehensive evaluation, and follow-up and patients would benefit from effective treatments in time.

## 1 Introduction

In December 2019, a succession of pneumonia cases, which were later proven to be caused by a novel coronavirus (named as novel coronavirus pneumonia-19 [COVID-19] by the World Health Organization [WHO] on January 12, 2020), emerged in Wuhan City, Hubei Province, China. In the following days, COVID-19 had quickly spread inside of Hubei Province, throughout China and other countries. A global health emergency was declared by the WHO on January 30, 2020 [1,2]. As of March 23, 2020, a total of 3,49,828 COVID-19 cases had been reported in at least 172 countries. Among them, 81,691 cases came from China and 2,68,137 cases came from the other countries. A total of 15,336 patients have died of COVID-19. Regarding the virus itself, the International Committee on Taxonomy of Viruses has renamed the previously provisionally named COVID-19 as severe acute respiratory syndrome coronavirus-2 (SARS-CoV-2) [3]. In the absence of specific therapeutic drugs or vaccines for COVID-19, it is critical to diagnose the disease at an early stage and immediately isolate the infected cases from the healthy population. Chest computed tomography (CT) is used for diagnosis of COVID-19, as an important complement to the reverse-transcription polymerase chain reaction (RT-PCR) tests [4]. Here, we reported two cases and the purpose of the study was to discuss the combination of chest CT and clinical features for diagnosis of COVID-19.

## 2 Case presentations

### 2.1 Case one

**Figure 1:**
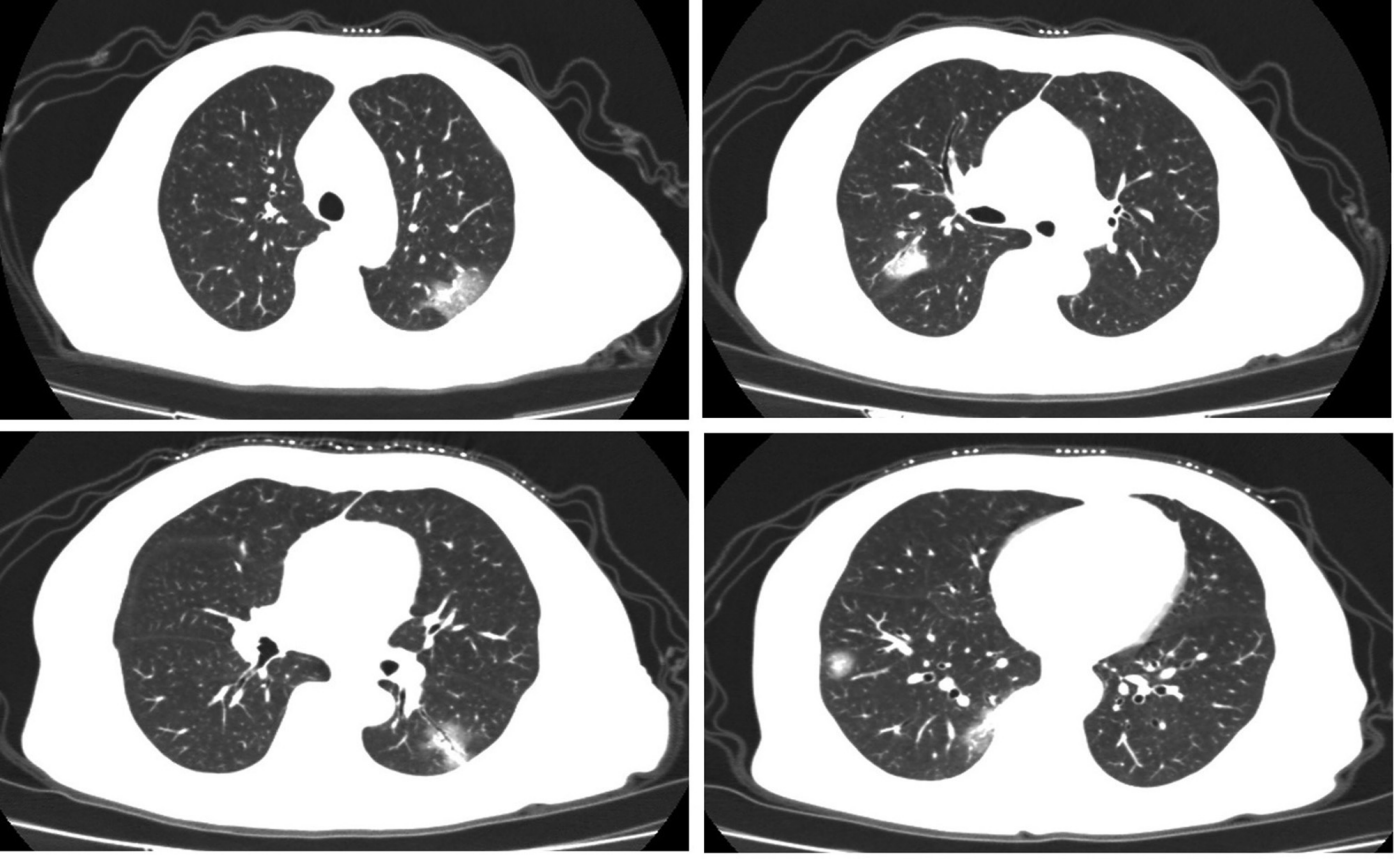
Case 1: CT chest result showed multiple peripheral solid and ground-glass images on bilateral lungs.

A 66-year-old female presented to our hospital with a 3-day history of fever with coughing, white sputum, runny nose, and dizziness. Two days before, her daughter-in-law was diagnosed with COVID-19. Her body temperature was elevated to 38.6°C. She was examined for complete blood count, C-reactive protein, and chest CT. The white cell count was 3.88  ×  109/L (reducing, range 4–11  ×  109/L), with low lymphocytes at 1.24 ×  109/L and normal neutrophils. The C-reactive protein was 10 mg/L (slightly elevated, normal  <  3 mg/L). Chest CT showed multiple peripheral solid and ground-glass opacities in both lungs (Figure 1). She was diagnosed with COVID-19, based on the positive nucleic acid test using swabbed samples from her throat and sputum.

### 2.2 Case two

**Figure 2:**
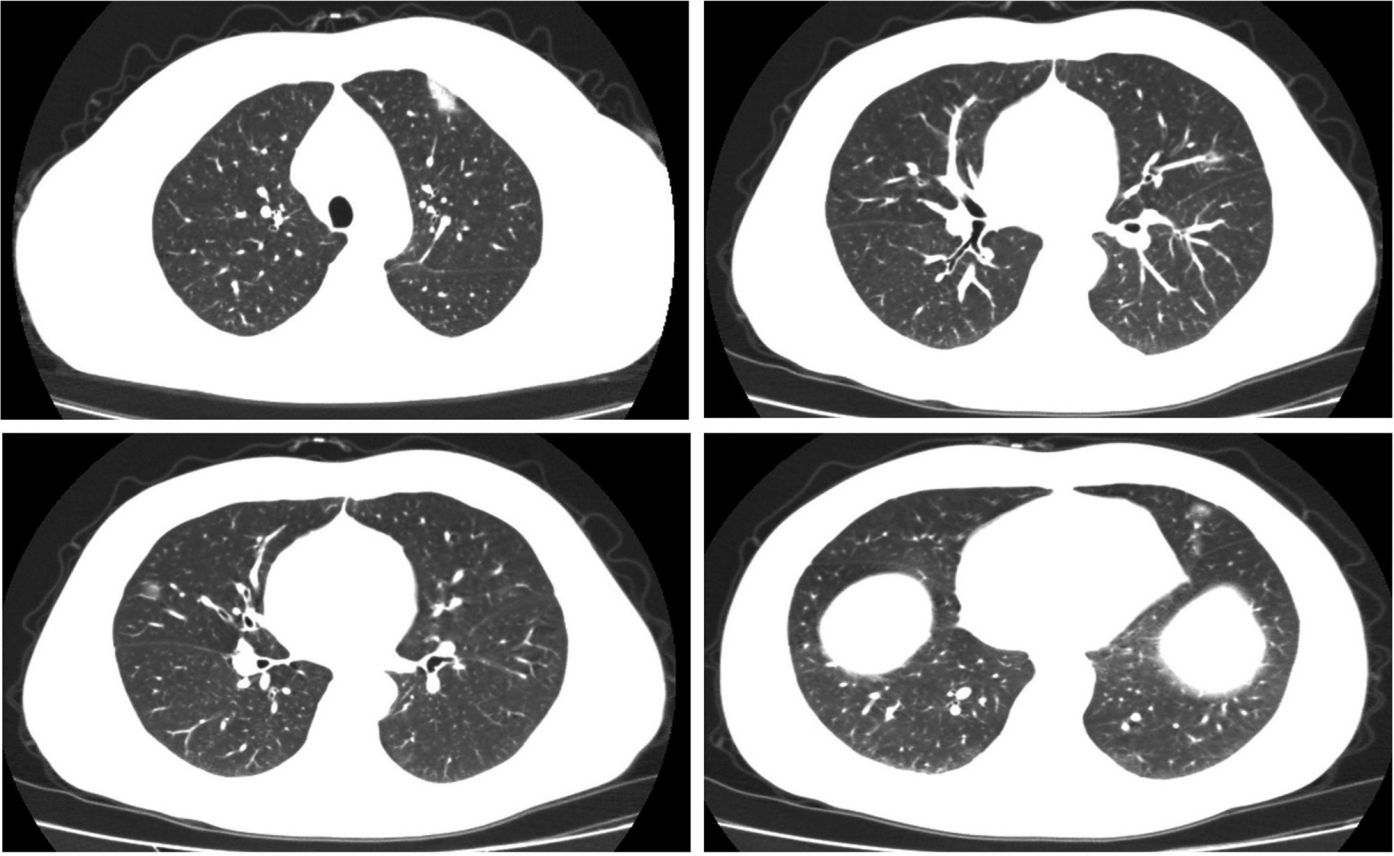
Case 2: CT chest result showed multiple peripheral ground-glass images on bilateral lungs.


A 50-year-old male presented to our hospital with a 2-day history of fever and dry cough. He denied having been to Wuhan. His body temperature was elevated to 38.5°C. The examination of complete blood count was normal but the C-reactive protein was slightly elevated (6.39 mg/L, normal <  3 mg/L). The chest CT result showed multiple peripheral ground-glass opacities on bilateral lungs (Figure 2). COVID-19 nucleic acid tests were repeated up to seven times using the patient’s throat samples and the test results remained controversial. The results from our hospital showed negative, positive, negative, and positive and the results from Jinan Center for Disease Control and Prevention (CDC) showed negative, negative, and positive. Eventually, he was diagnosed with COVID-19 pneumonia five days after initial symptoms.

**Ethical approval:** Jinan Central Hospital Institutional Review Board approved this study and written informed consent was given by the patient. The images are published under agreement of the patient.
**Consent for publication:** All the authors gave consent for publication.

## 3 Discussion

The epidemiological history is essential and very important to a kind of contagion, especially to diagnosis in early stage. Most patients outside Wuhan had been to Wuhan or had close contact with individuals from Wuhan. Guan et al. reported 1,099 COVID-19 cases from 552 hospitals in 30 provinces, autonomous regions, and municipalities in China through January 29, 2020. They found that 43.9% of patients lived in Wuhan. Among the cases who lived outside Wuhan, 72.3% had contact with residents of Wuhan [5]. The epidemiological history from Wuhan is essential to diagnose the disease. According to WHO Interim Guidance, the epidemiological history of COVID-19 included: within 14 days before the onset of the disease, (1) the case had a travel history to Wuhan or its surrounding areas, or other communities with confirmed patients; (2) the case had contact with confirmed COVID-19 patients; (3) the case had contact with suspected COVID-19 patients (fever or respiratory symptoms) from Wuhan or its surrounding areas, or other communities with COVID-19 patients [6]. The Chinese government has taken several effective measures to stop the spread of the disease from Wuhan to the entire country. New cases are gradually decreasing in China.

Fever, dry cough, and fatigue are the most common presenting clinical symptoms of the COVID-19 infection. Some cases also have nasal congestion, runny nose, or other upper respiratory symptoms. About 20% of patients had severe dyspnea, and the death rate for COVID-19 in China is nearly 3% [5,7]. WHO Interim Guidance also described COVID-19 clinical features included: (1) Fever and/or respiratory symptoms; (2) bilateral ground-glass opacity, infiltrates, or lung consolidation on CT images; (3) early onset of normal/decreased white blood cell count or decreased lymphocyte count following a sign of pneumonia on chest images [6,8].

Chest CT is a very important method to diagnose COVID-19. Typical CT imaging manifestations include: (1) quantity (often more lesions); (2) dominant distribution (mainly subpleural); (3) density (mostly uneven, a paving stone-like change mixed with ground-glass density and interlobular septal thickening, etc.); (4) shape (large block, patchy, lumpy, nodular, honeycomb-like or grid-like, cord-like, etc). Among them, multiple, patchy, segmental, or sub-segmental ground-glass density shadows in bilateral lungs are the most common COVID-19 CT images. Some are accompanied by fine-grid or small honeycomb-like interlobular septal thickening. The high-resolution computed tomography shows a slight high-density and ground-glass change with fuzzy edge in the fine-grid or small honeycomb-like thickening of interlobular septa. The thinner the chest CT scan layers, the clearer the above imaging manifestations are displayed [4]. The differential diagnosis of COVID-19 is facing challenges since many other lung diseases can have manifestations of multiple ground-glass opacities, such as interstitial lung diseases, drug-related lung injury, and other lung influenza infections. Accurate detection of COVID-19 RNA is a diagnostic method close to the gold standard and could be strongly recommended.

In our study, the first case had a 3-day history of fever with coughing, white sputum, runny nose, and dizziness. The patient had contact with a confirmed case of COVID-19. Her white cell count and lymphocytes were low. The chest CT showed multiple peripheral solid and ground-glass opacities in both lungs, which is a typical COVID-19 CT imaging manifestation. She was diagnosed with COVID-19 by a positive nucleic acid test. The first case was a typical COVID-19 case and the diagnosis was relatively easy to make on the basis of typical epidemiologic characteristics, clinical manifestations, chest images, and the nucleic acid test result.

The nucleic acid test is very important to control the spread of the disease, especially for the patients with mild symptoms. They may have negative chest CT findings but with positive nucleic acid test results. However, the detection of COVID-19 nucleic acid using RT-PCR has some shortcomings: (1) immature development of nucleic acid detection technology; (2) the patient’s viral load is low (an undetectable viral load in the patient); (3) different manufacturers may lead to different detection rates; or (4) clinical sampling is improper. Fang et al. reported that the diagnosis sensitivity using chest CT image was greater than that using nucleic acid test (98% vs 71%, *p* < 0.001) in 51 confirmed COVID-19 cases [9]. Ai et al. summarized 1,014 COVID-19 patients, and their study showed 59% (601/1,014) of the cases had positive RT-PCR results, whereas 88% (888/1,014) had positive chest CT results. The sensitivity of CT in suggesting that COVID-19 was 97% was based on positive RT-PCR results [10]. It is necessary to enhance the sensitivity of the nucleic acid test. Recently, for some hard-to-diagnose cases, COVID-19 antibody is another reliable method to make diagnosis.

**Figure 3:**
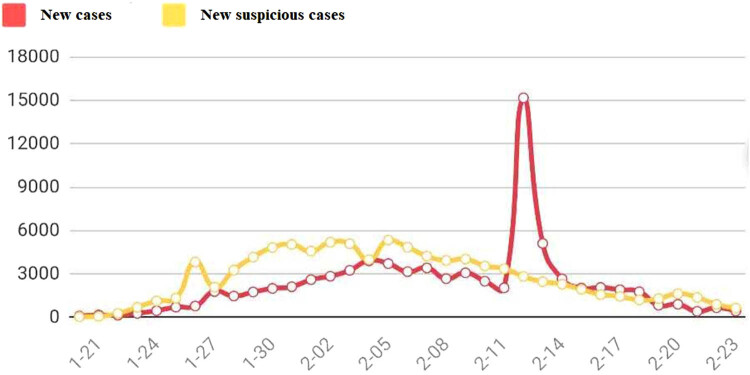
On February 12, 2020, a total of 15,152 new cases in China were confirmed COVID-19 on one day. Among of them, a total of 13,332 new cases in Hubei Province were clinically confirmed using chest CT without nucleic acid test.

It is important to detect COVID-19 at an early stage, and immediately isolate the infected cases from the healthy people due to a lack of specific therapeutic drugs or vaccines. It was reported that the total positive rate of RT-PCR for throat swab samples was about 30%–60% at initial presentation [11] with limitations of sample collection and transportation, and kit performance. In the current emergency, the low sensitivity of the nucleic acid test can lead to missed or delayed diagnosis and treatment of COVID-19 cases. Moreover, underdiagnosis of the infected cases can accelerate the spread of the virus due to its highly contagious nature. Chest CT is an easy, fast, and reliable diagnostic tool for a routine pneumonia diagnosis. It was reported that chest CT demonstrated pulmonary abnormalities consistent with COVID-19 in cases with initial negative RT-PCR results [12]. In our study, the second case had a 2-day history of fever and dry coughing. The chest CT result showed multiple peripheral ground-glass images on bilateral lungs, which is a typical COVID-19 CT imaging manifestation. He received COVID-19 nucleic acid tests up to 7 times with mixed results. The diagnosis of COVID-19 was delayed by five days. It is important to enhance the sensitivity of the nucleic acid test. Method of sample taking, quality control of the kits, and adequacy of the skills, all might affect the reliability of the nucleic acid test. On February 12, 2020, COVID-19 was first diagnosed clinically using chest CT in Hubei province according to “*diagnosis and treatment of novel coronavirus pneumonia in China (The Fifth Edition, passed on February 5, 2020)*”. A total of 13,332 new cases in Hubei province were clinically confirmed using chest CT without nucleic acid test in one day and timely treatments were provided. These infected cases were isolated immediately and more healthy people were protected (Figure 3).

## 4 Conclusion

In conclusion, our study shows that chest CT examination has high sensitivity for clinical diagnosis of COVID-19, particularly when the nucleic acid test result is negative. Chest CT should be considered as a useful tool for the screening, comprehensive evaluation, and follow-up of the COVID-19 cases, especially for determining the disease severity and monitoring the patient outcomes, therefore patients can benefit from effective treatment in time.

## Abbreviations

COVID-19: novel coronavirus disease-19
CT: computed tomography
RT-PCR : reverse-transcription polymerase chain reaction
SARS-CoV-2: severe acute respiratory syndrome coronavirus-2
WHO: World Health Organization
